# Evaluation of the Levonorgestrel-Releasing Intrauterine System Following Difficult Insertion in Perimenopausal Women: A Case Report

**DOI:** 10.7759/cureus.90186

**Published:** 2025-08-15

**Authors:** Kia Hui Lim, Jill C Lee

**Affiliations:** 1 Department of Obstetrics and Gynaecology, National University Hospital, Singapore, SGP; 2 Department of Urogynaecology, KK Women’s and Children’s Hospital, Singapore, SGP

**Keywords:** concealed lng-ius arms, difficult cannulation, ius complication, ius expulsion, mirena, missing lng-ius arms, perimenopause, transverse transvaginal ultrasound

## Abstract

The levonorgestrel-releasing intrauterine system (LNG-IUS) is a widely used long-acting reversible contraceptive, offering high efficacy and therapeutic benefits. Although generally safe, mechanical complications such as malposition, perforation, or retained components can occur. We report a case of concealed LNG-IUS arms in a perimenopausal patient following a difficult insertion, illustrating a rare but clinically significant complication.

Challenging insertions or removals, particularly in patients with a narrowed cervix or uterine anatomical changes, should raise suspicion for suboptimal device placement. When malposition is suspected, orthogonal two-dimensional (2D) transvaginal ultrasound views can improve visualization and guide management.

Awareness of arm retraction within the hormonal sleeve is essential to avoid unnecessary imaging or surgical intervention. Thorough device inspection, including sleeve dissection when required, should be the first step when missing arms are suspected.

This report emphasizes the importance of anticipating complications during difficult LNG-IUS insertions or removals and outlines practical diagnostic strategies for suspected malposition, maldeployment, or retained components.

## Introduction

Long-acting reversible contraceptives, including intrauterine devices (IUDs, hormonal and non-hormonal) and implants, account for 19.4% of global contraceptive use. Intrauterine contraception is utilized by 161 million women globally [1], with the hormonal option being the levonorgestrel-releasing intrauterine system (LNG-IUS), commonly known as Mirena (52 mg LNG-IUS, Bayer, Whippany, NJ). Mirena is a highly effective contraceptive, with a contraceptive efficacy of 99.3% for up to eight years [2], and provides notable therapeutic benefits. LNG-IUS has proven effective in managing menorrhagia [3] and dysmenorrhea [4] and is the first-line treatment for endometrial hyperplasia without atypia [5].

The LNG-IUS has a T-shaped body (32 mm × 32 mm), comprising two horizontal arms and one vertical stem. The soft and flexible T-shaped body is made of polyethylene compounded with barium sulphate, which makes it radiopaque and visible on X-ray. The vertical stem is wrapped with an elastomer sleeve, which consists of a mixture of polydimethylsiloxane and 52 mg of levonorgestrel. The system releases levonorgestrel over an extended period at a nearly constant rate, starting with an initial release rate of approximately 20 μg/day and maintaining a rate above 10 μg/day after five years [6].

Moreover, the IUD is uniquely associated with mechanical complications such as expulsion and perforation, requiring careful attention. Expulsion occurs in about one in 20 women, with risk factors including nulliparity, menorrhagia, severe dysmenorrhea, structural uterine anomalies, and insertions performed immediately postpartum [6-7]. Perforation, on the other hand, occurs in 2.6 per 1000 insertions, and the risk can be reduced by delaying insertion until at least eight weeks to six months postpartum [6-7]. Delayed detection of perforation may result in extra-uterine migration, causing adhesions, peritonitis, intestinal perforation, obstruction, abscesses, or erosion into adjacent viscera [8].

Currently, routine imaging to check the position of the LNG-IUS in utero is not performed immediately after its insertion. Instead, a vaginal examination is conducted to ensure that the retrieval threads are approximately 2 cm outside the external cervical os. However, if malposition is suspected, a two-dimensional (2D) sagittal transvaginal ultrasound with or without abdominal X-ray is commonly performed to locate the LNG-IUS and rule out mechanical complications. Further evaluation may include hysteroscopy or laparoscopy [7].

The LNG-IUS is also thoroughly inspected for integrity upon removal. Retention of part of the IUD can lead to complications such as pain, bleeding, pelvic infections [9], and continued contraceptive effects in cases of desired fertility [10]. Suspicion of a retained IUD would, therefore, warrant the aforementioned investigations to locate the device and rule out potential mechanical complications.

We present a case of concealed LNG-IUS arms in a patient with difficult cervical cannulation during insertion. This case highlights the importance of recognizing potential risk factors for suboptimal device placement or deployment, being vigilant about the possibility of concealed LNG-IUS arms, and checking for this to prevent unnecessary downstream investigations. However, if an IUD is truly retained, appropriate and adequate investigations should be conducted to confirm its presence and rule out complications.

## Case presentation

**Figure 1:**
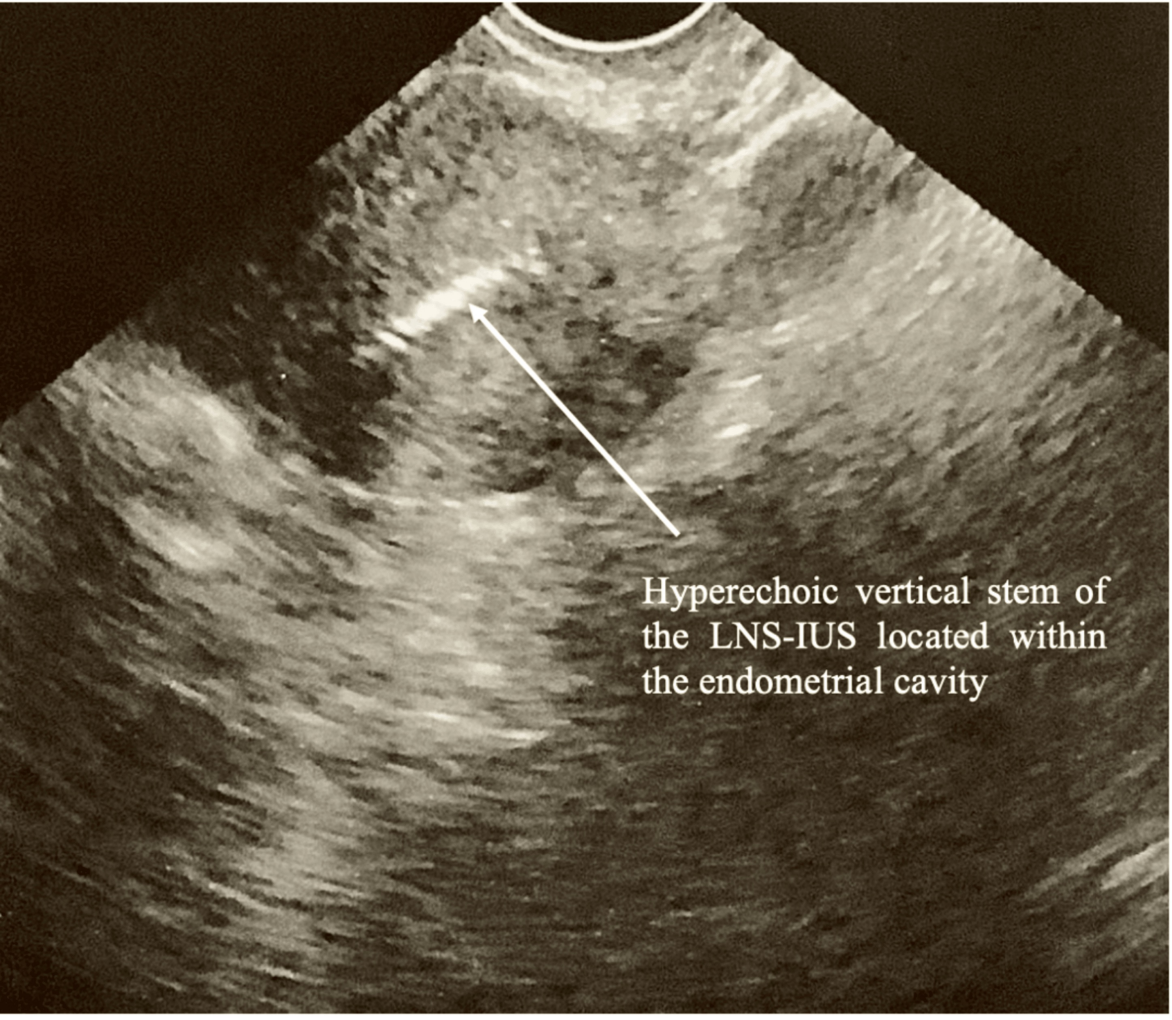
Two-dimensional (2D) transvaginal sagittal ultrasound confirming the presence of the vertical LNG-IUS stem within the endometrial cavity upon insertion. LNG-IUS, levonorgestrel-releasing intrauterine system

A 55-year-old, para 3 woman presented to the urogynecology clinic for the removal of her LNG-IUS, which had been inserted five years ago at the same institution for contraception. The bedside insertion was noted to be challenging and painful, requiring cervical dilatation with a size 5 Hegar dilator due to difficulty passing the sound through the cervix. This was a significant deviation from her previous multiple smooth Mirena insertions. Following the procedure, vaginal examination confirmed the presence of the LNG-IUS threads. However, concerns were raised about the potential for suboptimal placement of the LNG-IUS, including malposition, partial expulsion, or embedment in the myometrium, which could result from increased uterocervical manipulation or the additional force applied during insertion. To confirm the LNG-IUS placement post-insertion, a 2D sagittal transvaginal ultrasound was performed, which confirmed the presence of the vertical stem within the endometrial cavity (Figure 1). No complications were noted.

The patient was on yearly follow-up for a history of recurrent urinary tract infections, which did not recur since the insertion of her current LNG-IUS. She has a small right wall subserosal fibroid, which does not compress the endometrial cavity. Following insertion of the current LNG-IUS, her menstrual cycles were regular with light flow, with occasional irregular vaginal spotting over the first four years, as would be expected for a woman on LNG-IUS. No pelvic pain was reported, and she did not have any pregnancies during this time. She had been amenorrheic for a year at the time of removal. Routine transvaginal sagittal ultrasounds showed that the LNG-IUS stem remained in situ. On the day of the scheduled LNG-IUS removal, her abdomen was soft and non-tender on palpation. The LNG-IUS threads were visualized on speculum examination, and her cervix was noted to be atrophic. The LNG-IUS was then removed smoothly by grasping its threads with a pair of artery forceps.

**Figure 2:**
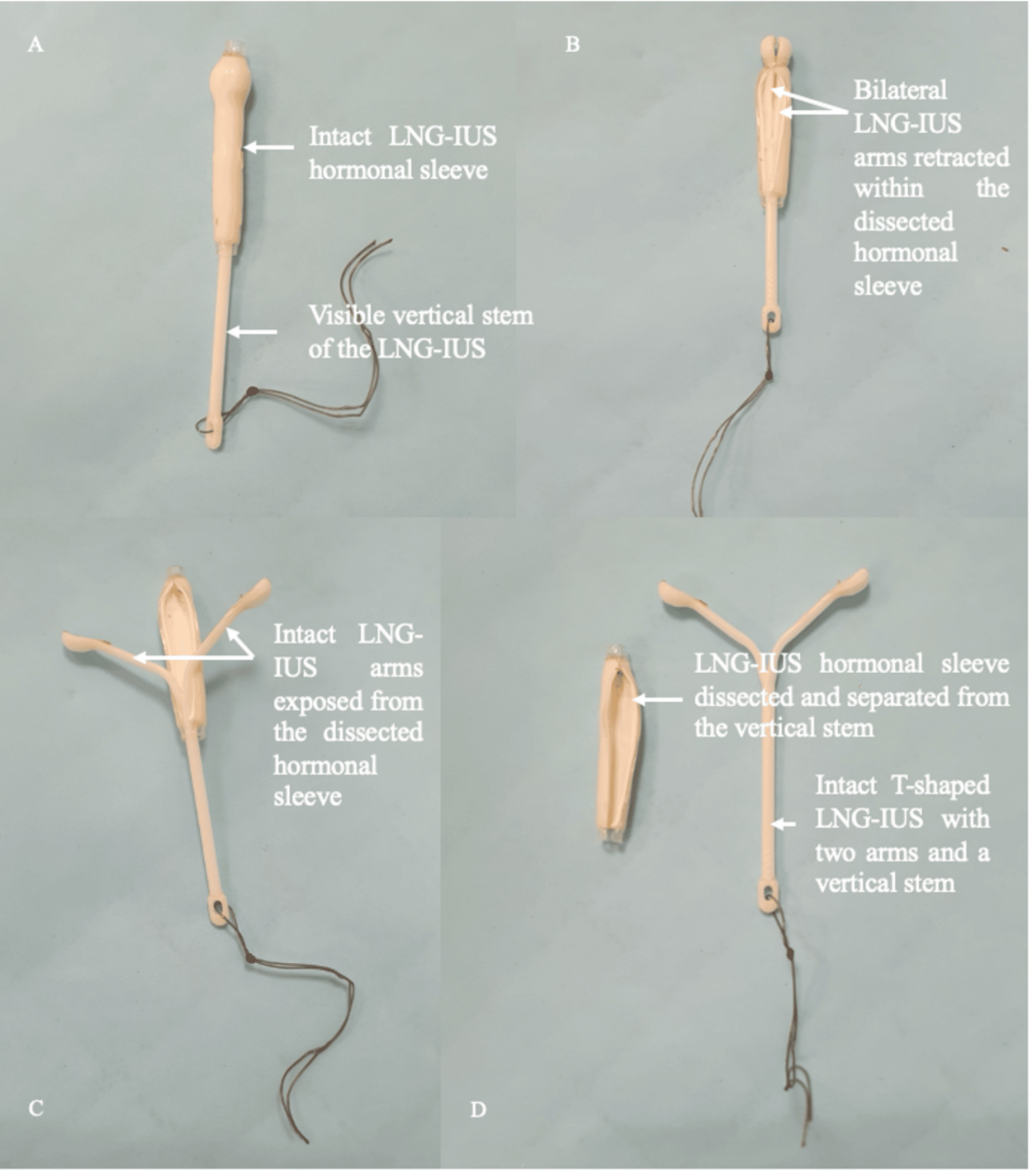
Close-up of removed LNG-IUS. (A) LNG-IUS with arms appearing to be missing bilaterally; (B) bilateral LNG-IUS arms found retracted or undeployed within the dissected hormonal sleeve; (C) bilateral LNG-IUS arms shown to be intact and exposed from the hormonal sleeve; (D) intact T-shaped LNG-IUS separated from the hormonal sleeve. LNG-IUS, levonorgestrel-releasing intrauterine system

However, upon removal, the arms of the LNG-IUS appeared to be missing bilaterally (Figure 2A). No broken LNG-IUS parts or adnexal masses were visualized on the bedside transabdominal and transvaginal sagittal ultrasound. To thoroughly investigate for any LNG-IUS breakage, a detailed transvaginal pelvic ultrasound and abdominal X-ray were ordered, in which no LNG-IUS parts were visualized. No abdominal pain or vaginal bleeding was reported by the patient at the one-month follow-up after LNG-IUS removal. Eventual dissection of the LNG-IUS hormonal sleeve revealed that the arms were retracted within (Figure 2B). It was then confirmed that the arms remained intact (Figures 2C, 2D).

## Discussion

Cases of concealed LNG-IUS arms within the hormonal sleeve discovered upon removal have also been previously reported. Torbe et al. [11] linked this phenomenon to difficult device retrieval, which required increased traction on the threads, leading to the hormonal sleeve migration during removal. Similarly, Bukowski et al. [12] attributed the concealment of the arms to possible retraction of the arms during removal due to cervical stenosis, which developed secondary to a loop electrosurgical excision procedure post-insertion. The dislocation or shifting of the hormonal sleeve over the LNG-IUS arms during challenging removals, such as through a tight cervical canal or surgical removal after perforation, has been reported in approximately seven per one million insertions [8].

In this case, the narrowed cervix presented significant challenges to cervical cannulation and LNG-IUS insertion. This may have contributed to device malfunction in several ways.

Excessive mechanical compression of the LNG-IUS device within the inserter, beyond the elastic limit of the arms or hormonal sleeve, could result in macro-level deformation or micro-level damage, affecting the device's shape memory. This, in turn, could impair the arms' ability to unfold correctly upon release into the uterine cavity, causing them to remain retracted within the hormonal sleeve.

Second, repeated handling and increased manipulation during the challenging insertion could have physically altered the device, such as bending or compressing the arms, which might impair their ability to deploy properly. Furthermore, excessive manipulation could induce material fatigue, reducing the device’s shape memory and contributing to arm deployment failure.

Third, at the time of removal, the cervix was noted to be atrophic due to menopausal changes. This atrophy could have caused the LNG-IUS arms to retract as the device passed through the further narrowed cervical passage.

The patient’s transition into menopause resulted in significant uterine cavity shrinkage, as evidenced by a decrease in uterine dimensions from 9.0 cm x 5.0 cm x 4.0 cm (a year before LNG-IUS insertion) to 4.9 cm x 4.0 cm x 2.7 cm (a month after removal) could have contributed to retraction of the LNG-IUS arms over time.

An overestimation of uterine depth during insertion or structural abnormalities of the uterus could have caused the arms to retract upon encountering resistance. There could also have been insufficient time given for the LNG-IUS arms to deploy. Nevertheless, the presence of a small subserosal fibroid and her anteverted uterus were unlikely to have interfered with the LNG-IUS insertion.

Inherent manufacturing defects in the LNG-IUS cannot be ruled out, as issues with the intrinsic release mechanism or abnormal flexibility of the arms may have led to faulty deployment. Compromising the device before its expiry date could affect its shape memory, preventing proper arm deployment. Unfortunately, a limitation of the LNG-IUS is that it cannot be reloaded into the inserter once released [8], preventing the integrity of the device from being tested before insertion. Without the anchoring of its arms against the uterine wall, the LNG-IUS is theoretically at a greater risk of migration or expulsion, compromising its efficacy and safety. Notably, there have been no reported cases of LNG-IUS system malfunctions, such as failure of the intrinsic mechanism to deploy the arms. This underscores the urgent need for the manufacturing company to conduct thorough investigations into the durability, quality, and long-term integrity of the device to ensure its reliability and safety.

While the concealment of LNG-IUS arms within the hormonal sleeve is a rare complication, this case sheds light on the importance of remaining vigilant to this possibility. A thorough inspection for any missing parts upon IUD removal should include dissection of the hormonal sleeve if applicable, as this is the most cost-effective approach, avoiding the need for additional investigations. 

This case also highlights the need to recognize a narrowed cervix as a potential risk factor for difficult insertion or removal, which can lead to suboptimal device deployment or placement. In such cases, further investigations may be required to determine the IUD’s position and rule out complications.

If malposition, maldeployment, or retained IUD parts are suspected and an ultrasound scan is warranted, relying solely on the typical 2D sagittal transvaginal ultrasound view may be insufficient. While it can evaluate IUD placement and potential complications, it cannot visualize the IUD arms, as it only captures the vertical stem of the LNG-IUS. When applicable, an additional 2D transverse view may be performed to identify the bilateral LNG-IUS arms, which have echogenic proximal and distal segments accompanied by posterior acoustic shadowing [13]. An abdominal X-ray is essential for locating the IUS when it is not visible on ultrasound, as the barium sulphate in the arms enhances detection on X-ray but not on ultrasound. Additionally, a three-dimensional (3D) coronal ultrasound of the endometrial cavity enhances IUS visibility and provides better positional clarity with the added volume dimension, making it valuable in cases of uncertainty [14-15]. Hysteroscopy, which is both diagnostic and therapeutic, may also be necessary if all imaging methods fail [7].

That said, the choice of imaging method, if required, cannot be generalized to all complex LNG-IUS cases. Patient anatomy may vary, influencing the preferred imaging modality. For example, 3D imaging may be preferred in patients with uterine anomalies [15]. Therefore, individualized approaches and appropriate investigations must be tailored to the clinical scenario and the resources available in different institutions.

## Conclusions

Given the widespread use of the LNG-IUS, it is crucial to understand its potential complications and the strategies for their investigation and management. Although rare, with an estimated incidence of 7 per one million insertions, the concealment of LNG-IUS arms is a complication that must be recognized. Thorough inspection of the device, including dissection of the hormonal sleeve, if necessary, should be the first step when missing arms are noted upon removal, as this is the most cost-effective first-line approach.

A narrow cervix, which can contribute to difficult insertion or removal, should raise suspicion for suboptimal device deployment or placement. In such cases, further investigations may be necessary to confirm the IUD’s position and rule out complications. When malposition or maldeployment is suspected, 2D orthogonal transvaginal ultrasound views are essential to visualize the IUD arms, as the typical sagittal plane may be insufficient.

With various imaging modalities available, it is important to tailor investigations effectively to specific clinical scenarios, considering the patient’s anatomy, the most likely differential diagnoses, and the optimal use of available resources, which may be limited in some institutions.
